# Caregiver awareness of reproductive health issues for women with intellectual disabilities

**DOI:** 10.1186/1471-2458-11-59

**Published:** 2011-01-28

**Authors:** Lan-Ping Lin, Pei-Ying Lin, Shang-Wei Hsu, Ching-Hui Loh, Jin-Ding Lin, Chia-Im Lai, Wu-Chien Chien, Fu-Gong Lin

**Affiliations:** 1 Graduate Institute of Life Sciences, National Defense Medical Center, Taipei, Taiwan; 2 School of Public Health, National Defense Medical Center, Taipei, Taiwan; 3 Graduate Institute of Healthcare Administration, Asia University, Taichung, Taiwan; 4 Department of Family and Community Medicine, Tri-Service General Hospital, Taipei, Taiwan; 5 Medical Affairs Bureau, Ministry of National Defense, Taipei, Taiwan; 6 Office of Medical Service, Tri-Service General Hospital, National Defense Medical Center, Taipei, Taiwan

## Abstract

**Background:**

Limited attention has been paid to the issue of reproductive health as it affects women with intellectual disabilities, despite reproductive health being a vital issue in public health policy for women in the general population. This paper describes caregiver awareness of reproductive health issues relative to women with intellectual disabilities who are being cared for in welfare institutions in Taiwan.

**Methods:**

The study employed a cross-sectional, questionnaire-based study which recruited 1,152 caregivers (response rate = 71.87%) from 32 registered disability welfare institutions in Taiwan. We classified their understanding/awareness of reproductive health issues into four domains: menstrual (1) and menopause (2) issues, sex education (3), and reproductive health services (4). Each domain had five associated yes/no questions and the total score for the four domains was out of a maximum of 20. Data were analyzed using SPSS 15.0 software.

**Results:**

We found that most of the caregivers were familiar with matters concerning sex education, menopause, and reproductive health services, but they lacked adequate understanding of issues associated with menstruation in women with ID. Many aspects of reproductive health such as "menstrual pain", "age at menarche", "masturbation", "diet during perimenopause", and "publicly available reproductive health services" were issues in which caregivers lacked adequate knowledge and required further instruction. Logistic regression analysis revealed that female caregivers with a university degree, and those who had experience assisting with reproductive health care were more inclined to have higher reproductive health awareness scores than their counterparts.

**Conclusions:**

This study highlights that service providers should offer appropriate reproductive health education to institutional caregivers, and that more attention be focused on the personal experiences and concerns of intellectually disabled women in future research.

## Background

There is general consensus in the healthcare community concerning the need for defined policies and services aimed specifically at reproductive health for individuals with intellectual disabilities (ID). It is likely that such undertakings would be supported by society as a whole. Previous studies have shown that the sexuality of people with ID has often been stereotyped, with this group typically characterized as being childlike and asexual, invariably leading to a denial of their socio-sexual maturity and needs. Rodgers suggested that women with ID should have more control over the non-medical aspects of menstrual management, so that their individual needs can be met [1]. Generally, people with ID have conservative attitudes towards sexual intercourse and homosexuality, but they may become involved in personal intimate contact with persons with whom they are familiar [2]. Schupf and colleagues encountered a variety of reproductive health issues, such as the age-adjusted likelihood of menopause being twice as high in women with Down syndrome as in women with other forms of ID [3], and despite the ban on involuntary sterilization, it appears that many parents and caregivers of persons with ID still support sterilization as a form of contraception, especially for persons with severe ID [2]. Servais [4] reviewed previous scientific studies that assessed the expectations and support needs of persons with ID in terms of sexual health, and pointed out that hygiene management, gynecological care, prevention of unplanned pregnancy, sexually transmitted diseases, and abuse have been frequently identified as areas in which the presence of ID dictates specific support needs.

The issue of sexuality in individuals with ID is complex and, given their cognitive limitations, frequently depends largely on the actions of caregivers [5]. Many studies have highlighted that women with ID generally receive inadequate counseling to deal with their reproductive health care, and caregivers often lacked the competence to deal with these events when they occur [6,7]. However, caregivers are the front-line workers for individuals with ID; they play a vital role in the provision of reproductive health care to individuals with ID, and their attitudes no doubt affect the quality of service provided to this group. McCarthy and Millard [8] suggested that research is required to establish if there are any particular aspects of appropriate reproductive health care for people with ID, particularly with respect to the attitudes of their primary caregivers. The purpose of the present study therefore is to describe caregivers' awareness of reproductive health issues with respect to women with ID who are being cared for in welfare institutions.

## Method

A cross-sectional, questionnaire-based study named "Caregiver Perceptions and Health Education Strategies with respect to Menopause in Women with Intellectual Disability" was carried out. The study was given ethical approved by the Institutional Review Board of the Tri-Service General Hospital, National Defense Medical Center (Approval number: 098-05-032). A total of 267 institutions (totaling 8508 staff members), officially registered at the end of June 2009 under the jurisdiction of the Department of Social Affairs, Ministry of the Interior in Taiwan, were included in the study [9,10]. The study subjects were recruited by convenience sampling, where institutions were contacted by telephone and invited to join the study. Finally, 32 institutions (16% of the institutions contacted) agreed to participate in the study. The study population was composed of staff working in a caregiving role at these registered disability welfare institutions. Data was collected by a structured questionnaire (in Chinese) that was completed by the institutional caregivers. The survey questionnaire included an informed consent letter, the caregiver's demographic characteristics, and tested their understanding of reproductive health issues for women with ID. According to WHO Guidelines on Reproductive Health [11], reproductive health is a state of complete physical, mental and social well-being, and not merely the absence of reproductive disease or infirmity. Reproductive health deals with the reproductive processes, functions and system at all stages of life. In this study, we divided awareness of reproductive health into four domains: understanding of menstrual and menopause issues, sex education, and knowledge of reproductive health services. Each domain had five yes/no questions, with the scoring range for each domain being 0-5 and the score for the four domains for each respondent out of a total of 20. The questionnaire was specifically designed and, to improve its validity, was reviewed and revised by five experts in the fields of clinical medicine, public health, nursing, special education, together with welfare institute staff. Questionnaires were mailed to the institutions, and distributed to caregivers. Completed questionnaires were collected from December 22, 2009 through until February 28, 2010 inclusive. Upon receipt of the questionnaires, data were entered into a database and analyzed using SPSS 15.0 software.

## Results

Of the total of 1,603 questionnaires mailed to the staff of 32 institutions, 1,152 were returned, giving a response rate of 71.87%. The demographic characteristics of the respondents are shown in Table 1. Female caregivers accounted for 89.8% of the respondents. The average age of respondents was 39.77 ± 10.23 years (range = 20-66 years), with approximately two-thirds of them possessing college and higher degrees. The respondents had spent an average of 6.62 ± 5.97 years (range = 0.1-26 years) working in welfare disability. Most of the respondents were first-line workers, such as special educators (47.4%) and living assistants (19.8%).

**Table 1:** Demographic characteristics of caregivers

Variable	n	%	Mean ± SD (range)
Gender (n = 1152)			
Male	117	10.2	
Female	1035	89.8	
Age (n = 1105)			39.77 ± 10.23 (20-66)
< 40	526	47.6	
≧40	579	52.4	
Educational level (n = 1134)			
Junior high school and less	77	6.8	
Senior high school	320	28.2	
College	269	23.7	
University	443	39.1	
Master and doctorate	25	2.2	
Job category (n = 1120)			
Manager	58	5.2	
Administrative staff	67	6.0	
Social worker	102	9.1	
Nurse	42	3.8	
Special educator	531	47.4	
Vocational trainer	90	8.0	
Living assistant	222	19.8	
Others	8	0.7	
Years working in this setting (n = 983)			6.62 ± 5.97 (0.1-26)
≦5	554	56.4	
6-10	196	19.9	
11-15	130	13.2	
≧16	103	10.5	
Years working in disability setting (n = 991)			7.32 ± 6.29 (0.1-33)
≦5	506	51.1	
6-10	223	22.5	
11-15	139	14.0	
≧16	123	12.4	

Table 2 presents the results of caregivers' understanding of issues concerning reproductive health for women with ID in relation to menstruation, sex education, menopause, and preventive health services. In relation to their understanding of issues concerning menstruation (table 3), the mean score was 3.98 ± 0.96, with almost one fourth of the respondents being unfamiliar with issues concerning this domain of reproductive health (score < 4). In particular, respondents responded incorrectly to statements such as "menstrual pain is one of the symptoms of reproductive diseases" (36.7%) and "it is abnormal to menstruate before 16 years of age" (37.1%).

**Table 2:** Caregiver perceptions of reproductive health for women with ID

Items of perception	Correct responders n (%)
*Menstrual perception*	
Self medication is the best way to relieve menstrual pain (n = 1144) (R)	1098 (96.0)
Appropriate exercise is allowed during menstrual period (n = 1137)	1005 (88.4)
The large amount of menstrual flow in the first three days is normal (n = 1140)	996 (87.4)
It is abnormal to menstruate before 16 years of age (n = 1131)	711 (62.9)
Menstrual pain is a symptom of reproductive diseases (n = 1131) (R)	716 (63.3)
*Sex education*	
It is not a crime to have sex with children under 16 years of age (n = 1145) (R)	1104 (96.4)
It does not get pregnant to have one sex intercourse (n = 1145) (R)	1101 (96.2)
A vaginal douche after sex can avoid pregnancy (n = 1143) (R)	1000 (87.5)
Using condoms during sex intercourse can prevent getting STDs (n = 1136)	920 (81.0)
Masturbation will cause impotence or frigidity (n = 1137) (R)	893 (78.5)
*Menopause perception*	
Hormonal fluctuations will cause physical or mental issues during perimenopause (n = 1139)	1103 (96.8)
Menopause is a natural process and not a disease (n = 1146)	1098 (95.8)
Women can adjust or stop hormone replacement medication during perimenopause without consultation with their doctor (n = 1141) (R)	1083 (94.9)
Menopausal women face a high risk of osteoporosis (n = 1143)	1057 (92.5)
A high fat, low fiber and calcium diet are recommended during perimenopause (n = 1137) (R)	870 (76.5)
*Preventive health services*	
Monthly self breast exams are necessary to prevent cancer (n = 1147)	1089 (94.9)
Regular Pap smear tests are necessary even if you have PVC vaccinations (n = 1145)	1076 (94.0)
Regular Pap smear tests depend on the needs of women aged over 30 years (n = 1143) (R)	1011 (88.5)
There is a free breast mammography service available every 2 years for women aged 45-69 years (n = 1141)	820 (71.9)
There is a free health exam service available every 3 years for women aged 40-64 years (n = 1133)	761 (67.2)

Items followed by (R) are reverse scored.

**Table 3:** Total score of caregivers' perception by reproductive health domains

Total score distribution*	n	%	Mean ± SD (range)
*Menstrual perception *(n = 1103)			3.98 ± 0.96 (0-5)
0	10	0.9	
1	13	1.2	
2	46	4.2	
3	202	18.3	
4	480	43.5	
5	352	31.9	
*Sex education *(n = 1126)			4.4 ± 0.89 (0-5)
0	7	0.6	
1	15	1.3	
2	15	1.3	
3	102	9.1	
4	335	29.8	
5	652	57.9	
*Menopause perception *(n = 1117)			4.56 ± 0.79 (0-5)
0	7	0.6	
1	7	0.6	
2	13	1.2	
3	61	5.5	
4	265	23.7	
5	764	68.4	
*Reproductive health services *(n = 1125)			4.16 ± 1.02 (0-5)
0	5	0.4	
1	11	1.0	
2	65	5.8	
3	195	17.3	
4	291	25.9	
5	558	49.6	

*Total score: 20.

In relation to sex education for women with ID, 87.7% of respondents had a good understanding (score≧4) on the whole of issues in this domain (mean score was 4.4 ± 0.89). However, nearly one quarter of respondents (21.5%) thought "masturbation will cause impotence or frigidity". With regard to their understanding of issues concerning menopause, most of the respondents also showed good awareness (mean score was 4.56 ± 0.79), particularly with respect to issues such as "hormonal fluctuations in the body", "menopause is a natural process and will cause risk of osteoporosis", and "women should consult with the doctor who prescribed hormone replacement medication". Only one false statement concerning "a high fat, low fiber and calcium diet are recommended during perimenopause" was incorrectly identified by 23.5% of respondents. In the final domain that dealt with "reproductive health services", the average score was 4.16 ± 1.02 and more than three quarters of respondents stated that they were satisfied with their awareness of issues in this domain. Nearly all of the respondents recognized the importance of breast self-examination and Pap smear tests. However, many respondents were unfamiliar with the free, publicly available reproductive health services for women in the health care system, such as a breast mammography service enabling women aged 45-69 years to undergo a free mammogram every 2 years (29.1% of respondents unaware) and a health examination service every 3 years for adults aged 40-64 years (32.8% of respondents unaware). Taken together, the mean total score for the four domains was 17.08 ± 2.51 (range = 0-20), with 31.1% of respondents scoring less than 17 points (table 4).

**Table 4:** Total score of caregivers' reproductive health perception

Total scores	n	%	Mean ± SD (range)
Score distribution (n = 1051)			17.08 ± 2.51 (0-20)
0	1	0.1	
1-5	5	0.5	
6-10	14	1.3	
11-15	175	16.7	
16-20	856	81.4	
Score category (n = 1051)			
< 17	327	31.1	
≧17	724	68.9	

Table 5 shows the relationship between caregiver characteristics and the score received in the survey. Results of bivariate chi-square analyses showed that the respondent's gender (p = 0.001), educational level (p = 0.006) and job category (p = 0.005) had a statistically significant effect on reproductive health awareness scores. Table 6 shows analysis results comparing caregivers' health experience characteristics and their reproductive health awareness score, with the results showing the importance of factors such as "experience assisting with reproductive health care for women with ID" (p = 0.005), "perception of adequacy" (p < 0.001) or "satisfaction with publicly available reproductive health services for women with ID" (p < 0.001).

**Table 5:** Relation of caregiver characteristics and reproductive health perception score for women with ID

Variable		Total score		
			
	N	< 17; n (%)	≧17; n (%)	χ^2 ^(p value)
Gender	1051			10.191
Male		50 (44.2)	63 (55.8)	(0.001)
Female		277 (29.5)	661 (70.5)	
Age (years)	1013			1.255
< 40		149 (29.7)	352 (70.3)	(0.263)
≧40		169 (33.0)	343 (67.0)	
Job category	1025			7.754
Front-line worker		228 (33.8)	446 (66.2)	(0.005)
Non-front line worker		89 (25.4)	262 (74.6)	
Years working in current setting	902			0.675
< 7		153 (27.5)	403 (72.5)	(0.411)
≧7		104 (30.1)	242 (69.9)	
Years working in disability setting	910			0.14
< 8		155 (28.4)	390 (71.6)	(0.708)
≧8		108 (29.6)	257 (70.4)	
Marital status	1045			3.157
Unmarried		111 (33.1)	224 (66.9)	(0.206)
Married		184 (29.1)	448 (70.9)	
Other		29 (37.2)	49 (62.8)	
Educational level	1035			7.686
College or less		201 (34.4)	383 (65.6)	(0.006)
University or higher		119 (26.4)	332 (73.6)	
Perceived health status	1042			0.318
Healthy		12 (35.3)	22 (64.7)	(0.573)
Poor		310 (30.8)	698 (69.2)	

**Table 6:** Relation of caregiver health experience characteristics and reproductive health perception score for women with ID

Variable		Total score		
			
	N	< 17; n (%)	≧17; n (%)	χ^2 ^(p value)
Experience assisting with reproductive health care	1028			7.937 (0.005)
Yes		149 (27.1)	400 (72.9)	
No		169 (35.3)	310 (64.7)	
Perceived adequate reproductive health knowledge	1012			1.133 (0.287)
Inadequate		223 (29.5)	532 (70.5)	
Adequate		85 (33.1)	172 (66.9)	
Adequacy of public reproductive health services for women with ID	1035			15.225 (< 0.001)
Inadequate		225 (28.2)	572 (71.8)	
Adequate		99 (41.6)	139 (58.4)	
Satisfaction with public reproductive health services for women with ID	1033			14.717 (< 0.001)
Unsatisfied		171 (27.0)	463 (73.0)	
Satisfied		153 (38.3)	246 (61.7)	
Adequacy of institutional reproductive health services for women with ID	1034			1.051 (0.305)
Inadequate		147 (29.9)	345 (70.1)	
Adequate		178 (32.8)	364 (67.2)	
Satisfaction with institutional reproductive health services for women with ID	1031			0.036 (0.85)
Unsatisfied		89 (30.8)	200 (69.2)	
Satisfied		233 (31.4)	509 (68.6)	

A multiple stepwise logistic regression was conducted to examine factors affecting the caregiver's reproductive health awareness (low and high score groups, the cut-off point score being 17 points) for women with ID. The model revealed that the factors of the caregiver's gender, educational level, and experience assisting with reproductive health care were statistically significantly associated with high reproductive health awareness for women with ID (Table 7). Those caregivers who were female (OR = 1.751; 95% CI = 1.12-2.73), with a university degree (OR = 1.404; 95% CI = 1.03-1.91), and those who had experience assisting with reproductive health care (OR = 1.373; 95% CI = 1.02-1.84) were more inclined to have higher reproductive health awareness scores than their counterparts.

**Table 7:** Logistic regression of caregiver reproductive health perception for women with ID (n = 977)*

Variable	β	S.E.	OR	95%CI	p value
Constant	0.554	0.121	1.741		< 0.001
Gender					
Male			1		
Female	0.560	0.226	1.751	1.12-2.73	0.013
Job category					
Front-line worker			1		
Non-front-line worker	0.314	0.166	1.368	0.99-1.90	0.059
Educational level					
College or less			1		
University or higher	0.339	0.156	1.404	1.03-1.91	0.030
Experience assisting with reproductive health care for women with ID					
No			1		
Yes	0.317	0.151	1.373	1.02-1.84	0.036
Adequacy of public reproductive health services for women with ID					
Inadequate			1		
Adequate	-0.367	0.199	0.693	0.47-1.02	0.065
Satisfaction with public reproductive health services for women with ID					
Unsatisfied			1		
Satisfied	-0.263	0.177	0.768	0.54-1.09	0.136

*Divided into two groups: score < 17 and score≧17.

## Discussion

Health issues for people with ID include respiratory problems, gastrointestinal disorders, challenging behavioral problems, and neurological conditions. This group as a whole carries a greater burden of diseases/disorders and requires more health services and preventive health interventions than the general population [12-19]. However, this group is commonly overlooked in relation to health concerns involving sexuality, sexually transmitted diseases, and end-of-life decisions [20]. Cambridge [21] suggested that the rights of people with ID to access information and receive support for sexuality and sexual health should be put first. The present paper has described caregivers' reproductive health awareness toward women with ID who are being cared for in welfare institutions. The results showed that most of the caregivers were familiar with sex education, issues of menopause, and preventive health services (mean score≧4), but they were unfamiliar with issues concerning menstruation (mean score < 4) in women with ID. However, many reproductive perceptions such as "menstrual pain", "age at menarche", "masturbation", "diet during perimenopause", and "free reproductive health services" were issues in which deficiencies were noted and in which caregivers needed to be provided with further instruction.

Caregivers in ID services have a clear role in encouraging women to live healthily and to ensure that women get good access to primary healthcare. Swango-Wilson [22] considered that caregivers are important to the educational experiences of individuals with ID, especially in relation to sexuality and decision-making when responding to the sexually-oriented behavior of others. Many primary caregivers believe that people with ID lack the capacity to make informed decisions about their sexuality or about having more intimate sexual relationships [23]. However, attitudes toward the sexuality of people with ID appear to have become more liberal with the passage of time. To this extent though, parents and staff differed in their attitudes, with parents holding more conservative attitudes [24]. Yool, Langdon and Garner [25] examined the attitudes of staff toward the sexuality of adults with ID within a medium-secure hospital in the United Kingdom, with their analysis revealing that staff members generally held liberal attitudes with respect to sexuality and masturbation. However, with respect to sexual intercourse, homosexual relationships, and the involvement of adults with ID in decisions regarding their own sexuality, less liberal attitudes were detected.

The attitudes of parents and teachers towards parenting by persons with ID remain negative, and these attitudes may adversely affect the provision of competency-enhancing supports and services for parents with ID and their children [2]. The present study found that the factors of institutional caregiver's gender, educational level, and experience assisting with reproductive health care were significantly associated in logistic regression analyses with high scores for reproductive health awareness for women with ID. Compared to previous studies, McConkey and Ryan [26] revealed that staff with previous experience dealing with sexual incidents involving teenagers and adults with ID, felt that they could deal with such issues more confidently in future, as did staff working in residential services rather than day services. Karellou [27] found that younger and more highly educated people expressed more contemporary attitudes towards human sexuality and the sexuality of people with ID than did older respondents. Similarly, a relationship was found between the level of education and the presence of more contemporary attitudes. Drummond [23] found primary caregivers were identified as holding more open attitudes to sexuality for the person with ID for whom they were caring, and these open attitudes were significantly influenced by a number of factors including age, level of education, marital status and religious beliefs. However, Bazzo et al. [5] found that educational level and the role of caregivers did not produce differences in their attitudes towards the sexuality of individuals with ID. A significant difference emerged between those who worked in different institutional services for this group of people.

This study has several limitations that need to be acknowledged. First, the lack of information coming directly from women with ID; our data were self-reported by key caregivers and subject to potential recall bias. In addition, the study samples were recruited from purposive sampling rather than random selection, which did not represent the full population of institutional caregivers working with ID subjects in Taiwan.

## Conclusions

The present study describes the profile of caregivers' understanding of the reproductive health of women with ID, and should provide valuable information for further educational programs to service providers. We suggest that health authorities should initiate education programs to improve the reproductive health knowledge of caregivers appropriately. Such programs need to consider factors such as the caregiver's gender, educational level, and experience assisting with reproductive health care issues, these items being significantly associated with adequate reproductive health awareness of caregivers with respect to women with ID. In addition, further research into reproductive health for women with ID is required to more precisely describe each case's personal experience in seeking care, the types of care provided, and the appropriateness of care received. To this extent, Britton [28] argued that "information giving" is not appropriate for explaining the embodied experiences of reproductive health issues such as menstruation in women with ID themselves. This approach, focusing primarily on the personal experiences and concerns of women with ID, will be necessary in future research.

## Competing interests

The authors declare that they have no competing interests.

## Authors' contributions

LPL contributed to the study/questionnaire design, data collection, data analysis, and the writing of the manuscript. LPY, LCI and CWC helped the study design, data collection and the analysis of the findings. SWH, CHL and LFG participated in the study design and interpreted data. JDL had a role in conceptual framework, coordination, manuscript writing and revision. All authors read and approved the final manuscript.

## Pre-publication history

The pre-publication history for this paper can be accessed here:

http://www.biomedcentral.com/1471-2458/11/59/prepub
